# Potentially fatal new trend in performance enhancement: a cautionary note on nitrite

**DOI:** 10.1186/1550-2783-7-25

**Published:** 2010-06-29

**Authors:** Andrea Petróczi, Declan P Naughton

**Affiliations:** 1 School of Life Sciences, Kingston University, Penrhyn Road, Kingston upon Thames, London KT1 2EE, UK

## Abstract

**Background:**

Considerable interest has been shown by athletes and scientists in the potential for nitric oxide and associated vasodilators to enhance performance. This study aims to explore potential misuse of vasodilators by the athletes, and to highlight the growing concern over these agents.

**Methods:**

Retrospective analyses of anonymous inquiries recorded in the Drug Information Database™ (DID™) between January 2006 and June 2008 (inclusive). In this 30-month period, the DID™ recorded 198,023 inquiries, of which 118,724 were UK Licensed Pharmaceutical products with a further 79,299 inquiries made for substance not found in the database.

**Results:**

Phosphodiesterase type 5 (PDE-5) inhibitors, dominated by Viagra^®^, ranked 16^th ^among the substance groups. The proportion of the inquiries made regarding PDE-5 inhibitors, especially in comparison to antibiotics, painkillers or alcohol, appears to be above the level that would normally be expected from medical need. No significant change in the months leading up to the Beijing Olympics was observed. On the contrary, the Nitric/Nitrate group showed a notable increase between 2006-2007 and 2008, suggesting a potential increase in interest in using nitric oxide among athletes.

**Conclusions:**

With patents recently filed for the use of agents containing sodium nitrite/nitrate to enhance blood flow for performance enhancement in sport, coupled with anecdotal evidence from internet athlete forums and media, there is a concern that athletes may endanger their health by using vasodilators to enhance athletic performance. PDE-5 inhibitors or chemicals in the nitrate/nitrate group are currently not prohibited or tested for by the doping control agencies but some are highly dangerous to health and can lead to cardiovascular collapse, coma and death. Its promotion among athletes as a performance enhancing supplement is ethically and medically questionable.

## Introduction

The discovery of the vasodilator role of nitric oxide (NO^·^) has led to a revolution in pharmacology over the past two decades which has brought considerable innovations in NO^·^-related therapy. Apart from helping to elucidate the mode of action of well established treatments such as nitroglycerine, the contribution of advances in NO^· ^research have mainly exerted an effect in the clinic through advances in the understanding and application of nitrite, a precursor to NO^·^. Just over a decade ago, the efficiency of NO^· ^production by the metallo-enzyme xanthine oxidoreductase was demonstrated [1]. In vitro and under hypoxia, this enzyme is considerably more effective than nitric oxide synthase at generating NO^· ^[1]. More recently, this phenomenon was observed for deoxyhaemoglobin [2], leading to the recent demonstration that nitrite has considerable protective effects in a range of cardiovascular conditions, including myocardial infarctions [3]. Nitrite, currently licensed for the treatment of cyanide toxicity, will undoubtedly continue to make a major clinical impact unless a serious side effect emerges. The long term benefits and risks of nitrite therapy have yet to be elucidated although *Martindale: The Extra Pharmacopoeia *lists the serious side effects as convulsions, cardiovascular collapse, coma and death.

Participants in professional sports can exhibit adept abilities to take advantage of advances in medicine relating to performance enhancement. It is not surprising that many athletes have looked at vasodilators to embellish their performances on the playing field. Reports of reliance on vasodilator drugs used for sexual dysfunction are common, even at the national team level. One report identifies the distributions of Viagra^® ^to a national soccer team playing at high altitude, supposedly without the players' knowledge [4]. This use has also been recognised by sport governing bodies as the World Anti Doping Agency (WADA) currently sponsor a study of the performance enhancing effects of sildenafil (Viagra^®^) at mild altitude [5].

**Figure 1:**
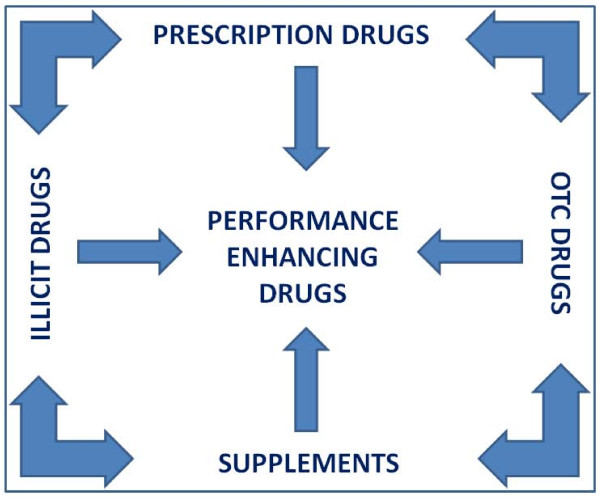
**Classes of drugs based on legal status**.

With the advent of easy availability of drugs and supplements via the internet, along with numerous unregulated discussion sites, it is concerning that athletes may unknowingly transgress from using harmless supplements to prescription only medicines in the absence of clinical supervision (Figure 1). The requirement for clinical supervision is reflected by the serious side effect profiles that are associated with these drugs. Our previous research shows that a concerning lack of understanding in supplements and their effect exist even among high-performing athletes who benefit from readily available support from nutritionists, doctors and physiotherapists [6-8]. Furthermore it has been shown that those who use supplements tend to use more than one concomitantly [8-12], including different types [13-15] and may move from one category to the next more effective substance [16-18]. As shown in Figure 1, various categories of substances willingly ingested by athletes and physically active people cannot be appropriately evaluated in isolation.

One approach to gauging the interests of athletes in vasodilators is to analyse inquiries lodged with the Drug Information Database™ (DID™). The DID™ was developed and hosted by elite sport^© ^and launched in the UK via UK Sport in 2002 and provided a self-check tool for athletes and support personnel (coaches, doctors, pharmacists, teachers, parents) until 2009. The anonymous inquiries were recorded since January 2006, cataloguing some 9,000 inquiries each month, predominantly from athletes themselves. The database contained UK licensed pharmaceutical products and was searchable by trade names and active ingredients, and linked to the current List of Prohibited substances published by the WADA [19]. Information returned on the individual inquiries included in- and out-of competition status of the drug, including differentiation by the route of administration.

The inquiries recorded via the DID™ have been scrutinized and shown to be a reflection of athletes' practices [20]. Beyond and above the evidence reported by Petroczi & Naughton [20], the validity of the database was further checked by mapping the trend in inquiries made about antihistamines and the onset of common pollen allergies (trees, grass and weeds). The close match between the inquiries and the seasonal variations in pollen allergies (data not shown) provided further reassurance for the validity.

**Figure 2:**
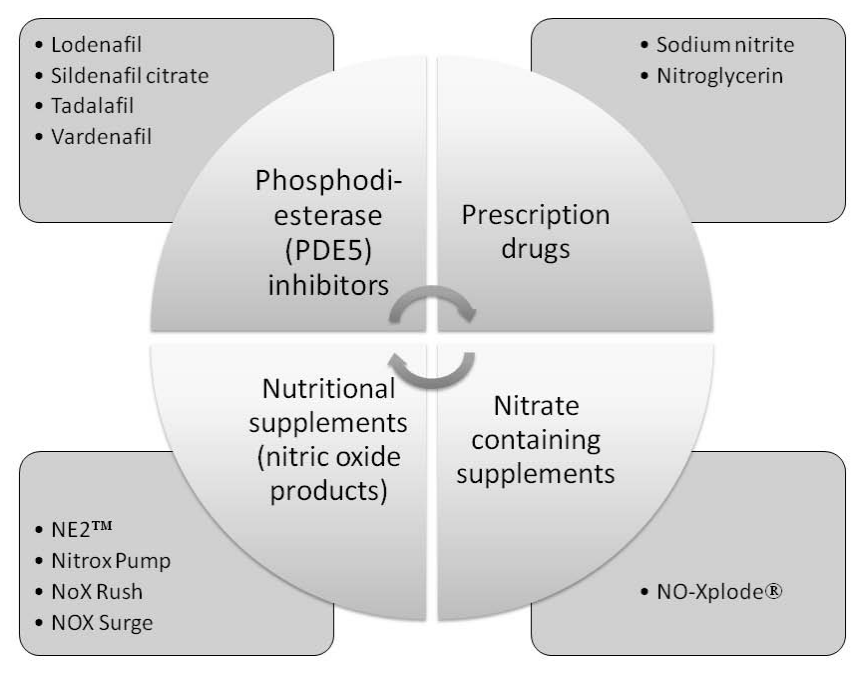
**Categories of nitric oxide-related compositions based on mechanism of action**.

This report presents an analysis of inquiries made on vasodilators to the DID™ during 2006-2008, leading up to the Beijing Olympics. It covers inquiries relating to i) prescription only phosphodiesterase inhibitors and ii) nitric oxide precursor supplement products. A key distinction between these two classes of vasodilator is their legal status with the former having been the subject of exhaustive clinical trials while the latter have been less well documented in terms of effects on human health. A further distinction is that the official standing of the prescription medicines affords the ability to study their use which is unavailable for the grey area of "nitric oxide precursor" supplement use. Additionally, it should be noted that the second class is comprised of supplement products of various compositions. Some of these are nitrogen-containing products that take advantage of the nitric oxide synthase pathway to form NO. Other compositions contain nitrites/nitrates (e.g., there is patent protection for a supplement agent containing sodium nitrite [21]). The differences within this class may not be apparent to many consumers, but the ingredients may have significantly different health or detrimental effects (Figure 2). Reports suggesting the use of prescription vasodilators to enhance athletic performance by professional athletes [4], may lead to an increased interest in prescription vasodilators in the sub-elite level of athletes leading to wider public health concerns. Furthermore, use of prescription vasodilators, whether obtained by prescription or not, may lead to the adoption of non-prescription nitrite supplements. For these reasons, it is timely to study the observed interest in these distinct classes of vasodilators in order to compare and contrast trends in interest by athletes. A key aim is to investigate fluctuations in the numbers of queries in each category, to establish if concerning trends in interest in the use of vasodilators has occurred over a two year period leading up to the Olympics.

## Methods

The UK Sport's Drug Information Database (DID™) has been previously interrogated to gain an insight into what substances athletes and their support personnel are interested in [20]. In order to elucidate the potential use or misuse of vasodilators, data previously downloaded from the DID™ were re-analysed. The data, limited to inquiries made in the UK, were downloaded in July 2008 in two segments:

• Dataset 1: Time covering between January 1, 2006, and December 31, 2007.

• Dataset 2: Six sets of 2008, in monthly segments, to monitor any changes during the months leading up to the 2008 Beijing Olympic Games. Comparing to previous analysis including all inquiries [20] and based on 2006-2007 (24 month period) statistics, enquires made in the UK formed the majority of the records (75% of "found" and 68.5% of "not found").

The present investigation focused on two major substance categories: Phosphodiesterase type 5 (PDE-5) inhibitors and the Nitric/Nitrate group. Keywords used to filter the data included both active ingredients and brand names (Table 1), but also included clearly identifiable word or phrase segments. Owing to widespread use of internet searches, we included drugs/brands that are not licensed in the UK. As the inquiries registered in the DID™ are recorded in two sets, those inquiries that relate to a drug or substance recognised by the DID™ ("found") and those that are "not found" [20], both sets were used. Statistical analyses were conducted using Excel XP^® ^version.

**Table 1:** Keywords used for analysing the DID™ data

Category	Dataset	Keywords
PDE-5 inhibitors	"Found"	Acetildenafil, Lodenafil (Helleva^®^), Microdenafil, Sildenafil citrate (Viagra^®^, Revatio^®^), Tadalafil (Cialis^®^, Adcirca^®^), Thiomethisosildenafil, Udenafil (Zydena^®^) Vardenafil (Levitra^®^, Vivanza^®^)

Nitrite/Nitrate	"Not Found"	Nitric oxide, Nitrate, Nitrix, NO2^®^, NO-Xplode^®^

In order to create meaningful groups of substances for further analysis, the recorded inquiries were ranked in decreasing order and individual contributions to the complete dataset were calculated and expressed as percentages. Previous analysis has shown that the number of inquiries recorded for each substance decreases exponentially with records greater than 200 yielding meaningful information [20]. A total of 45 substances received the highest number of enquires (> 300 for each substance), which individually account for at least 0.25% of the entire database. These popular substances were the focus of the analyses conducted for this report. Less popular formulations of these substances in the remainder of the database were also included. Queries about substances that match the database contents are listed as "found" whereas those that have no match are labelled as "not found". The latter category include those queries that were mistyped or do not correspond to a substance in the database. Thus, the "not found" category can provide information on emerging substances/names.

## Results and Discussion

The combined data (January 2006 to June 2008) contain 118,724 inquiries in the "found" dataset with the highest one being Lemsip preparations with 3,006 records (2.53%), followed by caffeine (2,045, 1.72%), ibuprofen (1709, 1.44%), paracetamol (1,648, 1.39%), ephedrine (1,440, 1.21%), and salbutamol containing preparations (1,235, 1.04%). Viagra^® ^was among the top 50 inquiries with 338 inquiries (0.28%). When combining all PDE-5 inhibitors, before grouping, there were 484 (0.41%) inquiries (equating to 25^th ^place) within this 30-month period.

**Figure 3:**
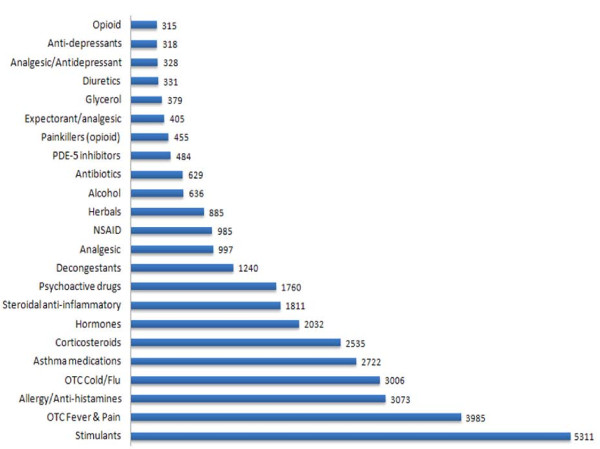
**Number of inquires grouped by class between January 2006-June 2008**.

Queries relating to substances with similar functions, such as stimulants or painkillers, can be grouped for clarity. The results were categorized by their main function and ranked as shown in Figure 3 for major categories. These categories equate to some 30% of all inquiries made. The breakdown was in the order: stimulants (4.5%); OTC fever and pain treatments (3.4%); allergy/anti-histamines (2.6%), for all queries made. Numbers of inquiries about PDE-5 inhibitors were on par with those about antibiotics, painkillers and alcohol. Given the population (young athletes), the proportion of interest in PDE-5 inhibitors appears to be above the level that would normally be expected for medical reasons. The main medical reason for such drugs, erectile dysfunction, in men below 40 years of age is very low (< 3%) [22] and only increases with chronic medical conditions (e.g. diabetes, severe obesity) or tobacco smoking - none of which is expected to be prevalent in the highly trained, competitive athlete group.

**Figure 4:**
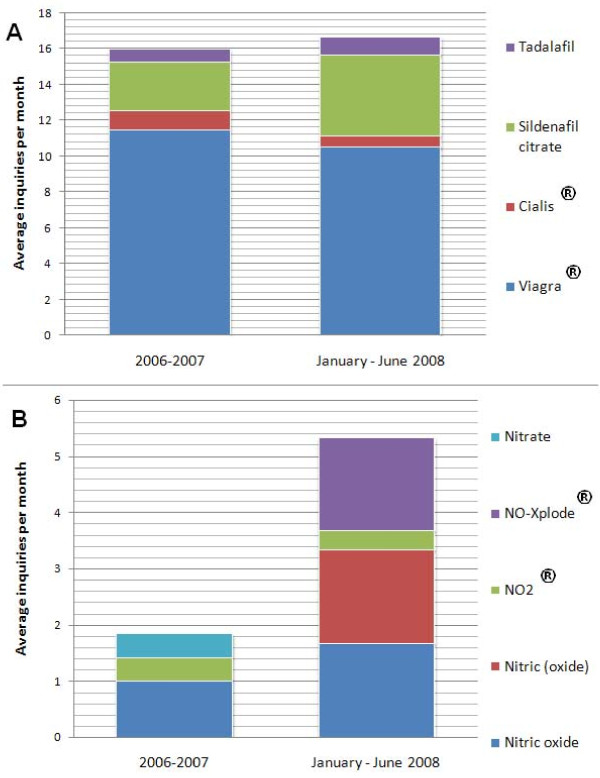
**Number of vasodilator related queries during 2006-2008 by category as A) "found" and B) "not found"**.

As shown in Figure 4, the total number of enquires about Viagra^® ^type substances per month is comparable between the two year period to 2008 and during the first six months of 2008. Among queries that match the database (i.e. "found") small shifts in numbers are seen in the latter period in favor of sildenafil and tadalafil, with minute losses against their brand names Viagra^® ^and Cialis^®^. A group of compounds identified as nitric oxide precursors were identified and monitored. These include (organic) nitrates, nitric, nitric oxide, NO2^® ^or NO-Xplode^®^. NO2^® ^appears on supplement distributor and bodybuilding web sites and is described as nitrite. In contrast, for nitric oxide related searches a three-fold increase in queries was observed despite the absence of these names on the database. In trends: the monthly average for the nitric/nitrate groups has steadily increased from 2.6% (2006) to 4.6% (2007) to 6.5% (2008). Thus, there has been a growing interest in nitrite related agents in contrast to a stable number of inquiries regarding Viagra^® ^type agents leading up to the Beijing Olympics.

Evidence from queries made to the DID™ along with sports internet discussion boards identifies a growing interest in blood enhancing agents including Viagra^® ^and nitric oxide based agents. A particular concern is the promotion of these drugs among athletes as performance enhancing supplements. Many athletes will be unaware of the potency and side effects associated with their abuse. In particular, sodium nitrite, the nitric oxide precursor, has led to fatalities. In a recent event, sodium nitrite was mistakenly used as salt for food preparation and led to two reported coma cases and four deaths [23], which highlights the toxicity at small doses that can occur outside of clinical supervision.

Many of the supplement products do not contain sodium nitrite, and it is not suggested here that the products on the market are themselves dangerous. However, agents to enhance blood flow for performance enhancement in sport have been subject to patent protection and in one case, the composition contains the active agents as sodium nitrite/nitrate [21]. The possibility that use of the other supplement products may lead to the use of dangerous products is the primary concern. Clearly, the clinical applications of nitrite are immense despite the potential drawbacks of, yet to be fully explored, therapeutic windows [3]. Recent reports of nitrite induced cardiovascular protection, based on proteome changes [24], have yet to be ascribed a mechanism. However, it is clear that oxidative damage occurs, as shown by the authors, which may elicit the protective effects leading to questions regarding long term use [24]. In recent years, there has been spreading speculation regarding the potential misuse of vasodilators by the athletic population [25]. PDE-5 inhibitors are currently not prohibited by the WADA but the agency has funded research to investigate the performance-enhancing potential of sildenafil [12].

Nitrite/Nitrate and related products are not on the WADA prohibited list of chemicals either; and as an endogenous species and component of foodstuffs a regulatory test is unlikely. From our current knowledge of doping reports, athletes are willing to use non-prohibited and OTC medications to boost their athletic performance [10-12]. It is concerning that these products frequently fall outside of medical supervision. Thus, a more acceptable policy is warranted, along with public awareness initiatives.

## Conclusions

This report demonstrates that, in contrast to interest in prescription vasodilators, athletes exhibited an increasing interest in "nitric-oxide precursor" vasodilators as observed in the DID™ records. There was a marked increase in inquiries made about these supplements leading up to the Beijing Olympics. Without medical supervision, use of vasodilators, especially (sodium) nitrite is potentially very serious and the adverse effects should be publicised.

## Competing interests

The authors declare that they have no conflict of interest.

Conclusions and recommendations made by the authors have arisen from the literature and the DID™ data. They do not necessarily represent the official position of UK Sport and should not be interpreted as such.

## Authors' contributions

The authors contributed equally with the inception and writing of the manuscript. Both authors read and approved the final manuscript.
